# Breast Cancer Pathological Image Classification Based on the Multiscale CNN Squeeze Model

**DOI:** 10.1155/2022/7075408

**Published:** 2022-08-29

**Authors:** Yahya Alqahtani, Umakant Mandawkar, Aditi Sharma, Mohammad Najmus Saquib Hasan, Mrunalini Harish Kulkarni, R. Sugumar

**Affiliations:** ^1^Faculty of Computer Science and Information Technology, Jazan University, Jizan, Saudi Arabia; ^2^SVKM'S Institute of Technology, Dhule, India; ^3^Department of Computer Science Engineering & Information Technology, Institute of Engineering & Technology (An Autonomous Constituent Institute of Dr. A.P.J. Abdul Kalam Technical University), UP, Lucknow, India; ^4^Wollega University, Nek'emtē, Ethiopia; ^5^Department of Pharmacy, School of Pharmacy, Vishwakarma University, Pune, India; ^6^Department of Computer Science and Engineering, Saveetha School of Engineering, Saveetha Institute of Medical and Technical Sciences, Chennai 602105, India

## Abstract

The use of an automatic histopathological image identification system is essential for expediting diagnoses and lowering mistake rates. Although it is of enormous clinical importance, computerized breast cancer multiclassification using histological pictures has rarely been investigated. A deep learning-based classification strategy is suggested to solve the challenge of automated categorization of breast cancer pathology pictures. The attention model that acts on the feature channel is the channel refinement model. The learned channel weight may be used to reduce superfluous features when implementing the feature channel. To increase classification accuracy, calibration is necessary. To increase the accuracy of channel recalibration findings, a multiscale channel recalibration model is provided, and the msSE-ResNet convolutional neural network is built. The multiscale properties flow through the network's highest pooling layer. The channel weights obtained at different scales are delivered into line fusion and used as input to the next channel recalibration model, which may improve the results of channel recalibration. The experimental findings reveal that the spatial recalibration model fares poorly on the job of classifying breast cancer pathology pictures when applied to the semantic segmentation of brain MRI images. The public BreakHis dataset is used to conduct the experiment. The network performs benign/malignant breast pathology picture classiﬁcation collected at various magnifications with a classification accuracy of 88.87 percent, according to experimental data. The diseased images are also more resilient. Experiments on pathological pictures at various magnifications show that msSE-ResNet34 is capable of performing well when used to classify pathological images at various magnifications.

## 1. Introduction

Breast cancer is the most common cancer in women [1], and the incidence tends to be younger. Pathological detection is regarded as the “gold standard” in the diagnosis of breast cancer [2], and pathological detection is determined by pathology. It is carried out under the microscope, and the pathological grade is given by the observation of the pathological section. Due to the large variability in the pathological images [3], the observer's experience and subjective differences may affect the most the final diagnosis. Benign tumors are noncancerous. They will not grow or invade surrounding tissue. However, when they form near vital organs, irritate a nerve, or restrict blood flow, they can be highly dangerous. The majority of benign tumors respond well to treatment. The only method to tell for sure is to get the lump biopsied, even though the fact that tests such as mammograms, ultrasounds, and MRI might provide hints as to whether a mass is malignant.

Malignant tumors are carcinogenic tumors. Our bodies constantly produce new cells to replace worn-out ones. Occasionally, DNA gets damaged during the process, resulting in abnormal cell formation. Instead of vanishing, they continue to expand at a rate that the immunity is unable to keep up with, leading to a tumor. Cancer cells can travel from the tumor to other parts of the body via the circulatory or vascular system. The location of the underlying tumor and whether it has spread are only two of the numerous variables that affect how malignant tumors are treated. Detailed information about the tumor can be revealed by a pathology report to aid with treatment planning, which may involve surgery, radiotherapy, chemotherapy, targeted therapy, and immunotherapy, commonly known as biological therapy.

Automatic classification algorithms for breast cancer pathological images can help pathologists make more accurate diagnoses. The research on breast cancer pathological image classification has made great progress in recent years, and the research methods for this task can be divided into There are 2 categories: one is algorithms based on manual feature descriptors and machine learning, and the other is algorithms based on deep learning.

Utilizing feature descriptors including binary patterns (lbp, gray-scale co-occurrence matrices, and classification techniques) such as random forests and support vector machines, the BreaKHis breast cancer pathology picture dataset [4] was produced. Using a majority vote method, literature [5] integrated the outcomes of each classification algorithm to get at an 87 percent detection accuracy for the same dataset. However, high-quality qualities require specialist knowledge and effort, limiting this technology's application.

Deep learning classification beats standard machine learning classification by adopting a network topology with the convolution layer as the core.

For the first time, literature [6] employs 11-layer and 13-layer deep neural networks to indicate the existence of mitosis in breast cancer histopathology pictures. In total, 14-layer convolutional neural networks have been utilized in the literature [7] to categorize breast pathology images as regular tissue, benign lesions, in situ carcinomas, or invasive malignancy. Literature [8] compared BreaKHis dataset studies utilizing AlexNet-based models. Machine learning categorization enhanced performance by 4–6%. Literature [9] used a magnification-independent deep network to acquire an 83% identification rate; Scheer vector and VGGNet's classification model provide an 87% recognition rate. The work requires preserving enough sturdiness for pathological photos at varied enhancements due to the significant differences in infected pictures at various magnifications. Deep network training needs many training examples, yet pathological breast cancer pictures are scarce. With the growing availability and incorporation of many data types, such as genomics, microarray, and histopathologic data, cancer therapy is moving toward precision medicine. It takes a lot of time and experience to use and analyze a variety of high-dimensional data formats for clinical or translational research jobs. Additionally, combining different data kinds requires more computing power than interpreting each type separately and calls for modeling algorithms that can absorb enormous amounts of complex characteristics. Machine learning algorithms are increasingly being used to automate these processes to help diagnose and detect cancer. Excitingly, DL models may be able to make use of this complexity to present insightful information and find pertinent granular characteristics from a variety of data formats.

To improve the classification model's effectiveness, maximize the few available samples. The channel recalibration model [10] focuses on feature channels. It suppresses superfluous features through learned channel weights and improves classification model performance. A multichannel CNN model was constructed and proposed as a solution to the identified problem using the sensitive lymph node pathologic imaging datasets for breast cancer. The model employs stacked multichannel convolutional units, Internet of Things-based CNN modules, skip cross-layer interconnections, a combination of classical and depth-wise separable convolution layers, and summation and concatenation operations. According to the results, the model does a good job of identifying micrometastases as well as lymph node metastasis.

This article is in the channel to enhance CNN feature utilization. A multiscale channel squeeze-and-excitation (msSE) model is developed according to the refinement model. It uses different max-pooling layers to gather multiscale features; channel recalibration is undertaken on each scale feature. The fused channel weights achieve multiscale channel recalibration for input characteristics. Multiscale features may improve the network's feature information, and channel recalibration can raise the classification model's performance. The network's training set includes breast cancer pathology images at four magnifications, guaranteeing the classification model is robust to multiple embellishments and meets clinical expectations.

## 2. Related Work

### 2.1. Residual Structure

One illustration of a multilayer neural network's unstable behavior is the vanishing gradient problem (VGP). Networks are unable to return gradient information to the model's input layers. Gradients for deeper layers in a multilayer network are calculated as the sum of many gradients of activation functions. These gradients will quickly disappear when they are tiny or zero. On the other hand, if they are more than one, it may explode. As a result, updating and computing become quite difficult. The partial derivatives for the variables of the NN, which are the gradient's constituent elements, grow exponentially tiny in the VGP, practically negligibly changing the variables with the gradient.

**Figure 1:**
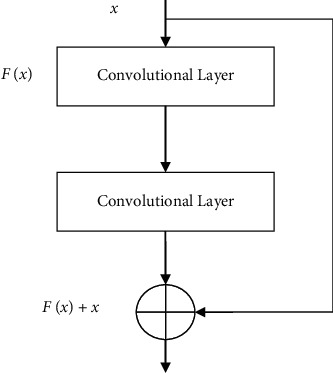
Residual structure.

The vanishing gradient problem of deep convolution neural networks makes it difficult to train deeper network models. The residual structure proposed literature [11] solves this problem and enables deeper convolutional neural networks to be trained efficiently. The structure of regression is shown in Figure 1.

The calculation process of the residual structure can be expressed as

(1) $$\begin{array}{c} y=F\left(x\right)+x, \end{array}$$

where *x* is the input feature of the convolutional layer, *y* is the output of the residual structure feature, and *F*(*x*) is the result after the convolutional layer mapping.

Suppose the residual structure is expected to fit the mapping as *H*(*x*). Due to the existence of the additional equivalent mapping, the mapping to be fitted by the convolutional layer in the residual structure becomes the mapping with residual (*x*)=*H*(*x*) − *x*. This is easier to learn than the original expected fitting mapping. The residual structure does not introduce additional parameters and can be trained through backpropagation. The residual network with the residual structure as the main body increases the number of network layers at the same time, which can avoid the gradient vanishing problem.

### 2.2. Channel Recalibration Model

**Figure 2:**
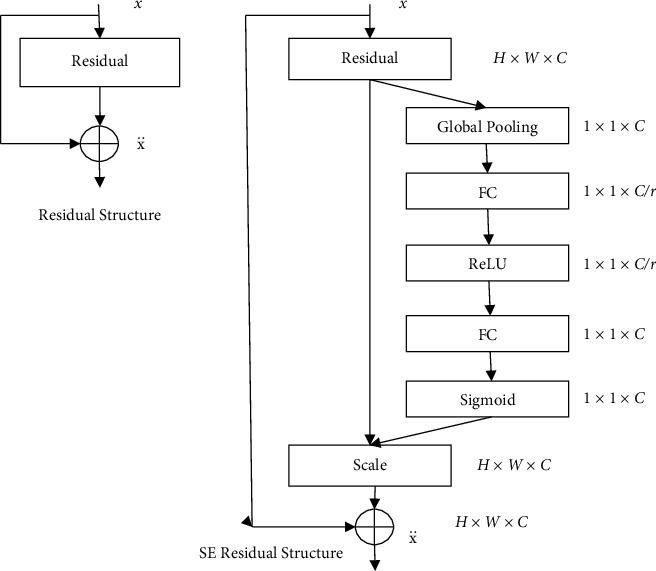
Residual structure and SE residual structure.

The attention model [12, 13] was first applied to natural language processing, by introducing attention weights to make the network model “attention” to useful In recent years, attention models have been applied to the field of computer vision [14, 15], by suppressing the uninteresting regions in the feature map, the network's attention is focused on the region of interest. Different from focusing on the feature map The channel refinement model is a model of attention acting on the channel domain of the feature map. It is proposed in SENet [10] designed by the ILSVRC17 competition classification task champion. The channel recalibration model weights the input features by channel, so that the network's attention is focused on useful features, and the channel weights can be learned through training. The channel recalibration model can be combined with VGGNet [16], ResNet [11], GoogLeNet [17], and other networks, and the residual structure and join The SE residual structure of channel recalibration is shown in Figure 2.

The channel recalibration model squeezes the input feature *U* in channel order according to:

(2) $$\begin{array}{c} z_{c}=F_{sq}\left(u_{c}\right)=\;\frac{1}{H\;\times W}\;\sum_{i=1}^{H\;}\sum_{j=1}^{W}u_{c}\left(i,j\right), \end{array}$$

where *z*_*c*_ is the result of squeezing the feature of the first channel in the input feature; *F*_*sq*_(*∗*) is the squeeze function; *u*_*c*_ is the feature of the first channel *c* in the input feature, and *H* and *W* are the height and width, respectively; *u*_*c*_(*i*, *j*) is the value of the feature at  *u*_*c*_  the spatial position (*i*, *j*). This process can be regarded as a channel-by-channel global pooling operation on the input features.

After the extrusion of each channel feature in the input feature is completed, the weight of each channel is obtained by exciting the extrusion result by the following formula:

(3) $$\begin{array}{c} s=F_{ex}\left(z,\;W\right)=\;\sigma \left(W_{2}\delta \left(W_{1}z\right)\right), \end{array}$$

where *s* is the weight of the feature channel; *F*_*ex*_(*∗*, *∗*) is the excitation function; *z* is the result of extruding the feature; *σ*(*∗*) is the sigmoid function; *δ*(*∗*)  is the ReLU function [18]; *W*_1_ and *W*_2_ are the weights of the two fully connected layers FC, respectively.

The first fully connected layer in the excitation process converts the number of feature channels depending on *c* reduced to *c*/*r*, where the compression ratio, and the output is only retaining values greater than zero after the ReLU function. The second fully connected layer restores the number of feature channels to *c*, so as to be consistent with the number of channels of the input feature. The final weight is obtained through the sigmoid function and the limit is 0∼1.0:

(4) $$\begin{array}{c} {\ddot{x}}_{c}=\;F_{\text{scale}}\left(u_{c},s_{c}\right)=\;s_{c}u_{c}. \end{array}$$

In the above formula: ${\ddot{x}}_{c}$ is the output characteristic of the channel after the recalibration of the channel feature; is the weight of the *c*^th^ channel in the input feature; *F*_scale_(*∗*, *∗*) is a scaling function, which is used to multiply the features of a specific channel with the corresponding channel weight. Equation (4) realizes the recalibration of the feature channel by multiplying the feature of a specific channel with the corresponding channel weight, and the whole process suppresses the features that are useless to the classification result, thereby improving the classification accuracy.

## 3. Proposed Algorithm Description

**Figure 3:**
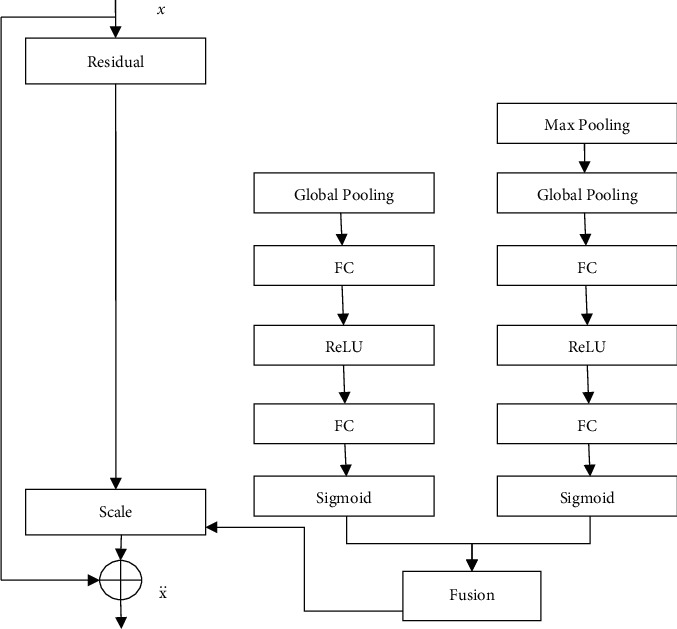
msSE residual structure.

On the basis of the channel recalibration model, the input of the channel recalibration model is changed from single-scale features to multiscale features, and the feature channel weights learned at each scale are fused to obtain the final feature channel weights. Add multiscale The msSE residual structure of the channel recalibration model is shown in Figure 3. Convolutional neural networks using multiscale features are often used in tasks such as target detection and recognition [15, 19–21] and image semantic segmentation [22–24]. Using feature information at multiple scales can make the final result more accurate. In Figure 3, the multiscale features are combined with spatial pooling gold.

Pyramid [19] similar structure is obtained: the input features are sent to the max pooling layer with a pooling kernel size of 2 × 2 and a pooling stride of 2 to obtain features of another scale. To obtain more scales, features can be achieved by changing the number of max pooling layers and related parameters. The reason for using max pooling layers to obtain multiscale features is that the max pooling operation can retain the most significant feature information and corresponding spatial information in the feature map; the maximum. The pooling layer has no model parameters that need to be learned and can achieve multiscale features while ensuring that as little computation as possible is introduced into the network. In Figure 3, fusion represents the fusion process of channel weights. Although channel attention appears to be economical in terms of variables and FLOPs overhead, one key problem is the scaling process, which involves broadcasting the weighted channel vector and applying by multiplying it element-wise to the input tensor. This intermediary broadcasted tensor occupies the same dimension as the input, resulting in a significant rise in memory complexity. As a result, the training process becomes slower and more memory intensive. The approach is highly expensive and augments the original model with a sizable number of parameters and FLOPS. Although in the big scheme of things, this overhead could be relatively little, there have been numerous novel ways that have outperformed SENets in terms of giving network attention at a very low cost. The method of maximum value and splicing fuses the feature channel weights obtained at different scales.

### 3.1. Additive Fusion

In Figure 3, the channel weight obtained by the additive fusion method 2 is the element-by-element addition of the channel weights under the two feature scales, and then the obtained weights are multiplied by the input features in the order of the corresponding channels to achieve multiscale channel recalibration. A process is as follows:

(5) $$\begin{array}{c} {\text{Ù}}_{2\text{way}\_\text{add}}=\;\left(S_{c0}+\;S_{c1}\right)\;U_{s0}. \end{array}$$

In the formula Ù_2way_add_ for 2 the results of multiscale channel recalibration using additive fusion at each feature scale, *U*_*s*0_ is the input feature, and *S*_*c*0_ is the channel weight of the input feature, *S*_*c*1_ is the channel weight at another scale.

### 3.2. Maximum Fusion

Unlike additive fusion, maximum fusion selects specific channels 2 the maximum value of the weight under each scale is used as the weight of the channel. At this time, the multiscale channel recalibration process is as follows:

(6) $$\begin{array}{c} {\text{Ù}}_{2\text{way}\_\text{max}}=\;\left(S_{c0},\;S_{c1}\right)\;U_{s0}. \end{array}$$

In the formula *Ù*_(2way_max) for 2, the results of multiscale channel recalibration using maximum fusion at each feature scale; max(*∗*, *∗*) is the maximum function.

Select 2, respectively, in channel order the maximum value of the channel weight under each scale is used as the weight of the channel.

### 3.3. Splicing Fusion

When there are two scale features, the splicing fusion method first splices the channel weights at each scale according to a specific coordinate axis, and then maps the result to the final channel weight through the subsequent convolution layer. The channel weight size is *N* × *C* × 1 × 1, where the batch image size is and the number of channels of the input feature is C The specific implementation of splicing fusion can be divided into the following two types according to the selection of the splicing coordinate axis.

(a) Take the second coordinate axis (axis1) as the splicing coordinate axis, denoted as cat1. At this time, the multiscale channel recalibration process can be expressed as
  (7) $$\begin{array}{c} {\text{Ù}}_{2\text{way}\_\text{cat}1}=\;F_{\text{conv}1}\left(S_{c\_\text{cat}1}\right)\;U_{s0}, \end{array}$$
where Ù_2way_cat1_ is the result of multiscale channel recalibration achieved by splicing and fusion cat1 at 2 scales, *S*_*c*_cat1_ is the result of splicing the channel weights obtained according to the second coordinate axis, size is *N* × 2*C* × 1 × 1, F_conv1 (*∗*) for the convolutional layer conv1 the mapping function, the size of the convolution kernel is 1 × 1,the number of input channels is 2C, and the number of output channels is C.
(b) Take the third coordinate axis (axis2) as the splicing coordinate axis, denoted as cat2. At this time, the multiscale channel recalibration process can be expressed as
  (8) $$\begin{array}{c} {\text{Ù}}_{2\text{way}\_\text{cat}2}=\;F_{\text{conv}2}\left(S_{c\_\text{cat}2}\right)\;U_{s0}, \end{array}$$

where Ù_2way_cat2_ is the result of multiscale channel recalibration realized by splicing and fusion cat2 at two scales, *S*_*c*_cat2_  is the result of splicing the channel weights obtained in the two scales according to the third coordinate axis, size is *N* × 2*C* × 2 × 1,  *F*_conv2_(*∗*) is the mapping function of the convolution layer, where the size of the convolution kernel is 2 × 1, The number of input and output channels are both *C*.

## 4. Experimental Results and Analysis

### 4.1. Dataset

**Figure 4:**
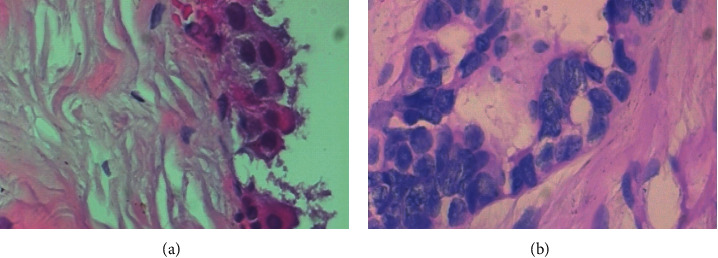
Benign and malignant breast tumor images. (a) Benign breast tumor image. (b) Malignant breast tumor image.

Experimental dataset: BreaKHis dataset, includes 7909 breast cancer pathology pictures from 82 individuals (24 benign and 58 malignant). The 700460 pathological photos in the dataset have 4 magnifications (40*x*, 100*x*, 200*x*, 400*x*).  Table 1 shows the dataset's picture distribution.  Figure 4 shows benign/malignant breast tumors from BreaKHis.

### 4.2. Experimental Environment and Settings

The accuracy Acc (accuracy), the precision rate Pr (precision), the recall rate *R* (recall), and the area under the ROC curve AUC are used as the measurement indicators of the classification results.

The formulas for calculating the rate and recall rate are as follows:

(9) $$\begin{array}{c} A_{cc}=\;\frac{TP+TN}{TP+FP+TN+FN^{\prime }},\\ P_{r}=\;\frac{TP}{TP+FP^{\prime }},\\ R=\;\frac{TP}{TP+FN}. \end{array}$$

In the above equation, TP represents the true positive example, FP represents the false positive example, and TN represents the true negative example, and FN means false negative example.

The dataset was not augmented in the experiment. The ratio of the training set to the test set was divided into 85% and 15%, and the images included were randomly selected at the beginning of the training. All comparative experiments used official source code or public code, all the network model uses the same image preprocessing method and training settings, and the experimental data of each network is obtained by averaging the results obtained by 5 times of training.

The training images are preprocessed as follows: (1) The image size is adjusted to a fixed 224 × 224; (2) The image is randomly rotated by 90°; (3) The brightness, contrast, saturation, and chroma of the image are randomly fine-tuned, which makes the training. The network can be more robust to the staining differences between pathological images; (4) normalize the images. Different from the training set, the preprocessing method of the test set images only includes adjusting the image size to 224 × 224 with normalization chemical processing.

The initial parameters of all network models in the experiment are obtained by random initialization, and the loss function is binary cross entropy, using momentum. The weights are updated using the network's stochastic gradient (SGD) algorithm. The momentum value is set to 0.9 and the starting training rate is set at 0.0001. The network's retraining batch image size is 64, whereas the test batch picture size is 128. Res-Net18-based network the number of training iterations is 10,600, and the number of network training iterations based on Res-Net34 is 21,200. If the test accuracy does not improve after every 1060 iterations, the learning rate is reduced to 0.1 times before.

Since the multiscale features are obtained by down-sampling the input features and the convolutional layer in the network will reduce the feature size, in order to keep the feature size in a reasonable range, the experiment only selects networks with feature scales of 2 and 3 for experiments. Select the network with 2 feature scales as msSE-ResNet-2way, and the network with 3 feature scales as msSE-ResNet-3way. In splicing fusion, the network that applies the sigmoid function to the output is added after its name adds sigma to differentiate.

### 4.3. Experiments Based on ResNet18

**Figure 5:**
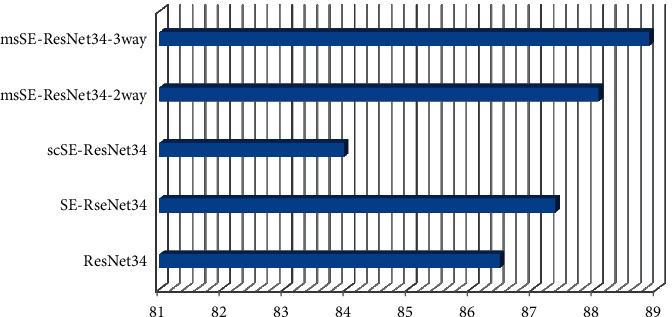
Comparison of accuracy between msSE-ResNet34 and other networks.

Comparison and analysis of msSE-ResNet18 and other networks, the experimental results of each network on the test set are shown in Table 2, and the ROC curve is shown in Figure 5. In the figure, FPR is the ratio of false positives, and TPR is true positives the classification results of breast cancer pathological images of different magnifications in the test set by each network in the experiment are shown in Table 3.  Table 4 shows the comparison of magnification-related classification results for all networks.

It can be seen from Table 2 that the test accuracy of ResNet18 is 84.53%, which is higher than the 83.56% of SE-ResNet18. Literature [16] proposed a spatial channel recalibration model (spatial and channel Squeeze-and-Excitation, scSE), which simultaneously performs spatial and channel recalibration, and use the maximum value of the weights obtained by the two as the feature channel weight. The accuracy of scSE-ResNet18 is 83.90%. The accuracy of msSE-ResNet18-2way reaches 86.81%, and the test of msSE-ResNet18-3way the accuracy is 86%, which is significantly improved compared with other networks. The comparison of magnification-related classification results for all networks is shown in Figure 4.

The ROC curve in Figure 5 further reflects the classification performance of each network. The AUC of msSE-ResNet18 with two scales is above 0.9, achieving better performance than other networks.

The experimental findings are shown in Table 3, and they show that msSE-ResNet18-2way retains strong resilience to diseased pictures at various magnifications; at magnifications greater than 40 times, msSE-Res-Net18-3way has the same performance as msSE-ResNet18-2way comparable classification performance. Since the task of the experiment is to classify benign/malignant breast pathological images, the classification accuracy is more important. The msSE-ResNet with multiscale channel recalibration is obtained under each magnification. The accuracy rate is higher than that of other comparison networks, which means that msSE-ResNet18 can more accurately find the malignant samples in the test set, achieve a high recall rate under the premise of ensuring high accuracy, and can find as many positive samples as possible.

The above experimental results show that the multiscale channel recalibration model can recalibrate the input features more accurately by combining the feature information at multiple scales and can improve the performance of the classification model while maintaining robustness to pathological images under different magnifications awesomeness.

Comparison and analysis of msSEResNet18 using different number of feature scales and fusion methods. The experimental results of msSE-ResNet18 using different number of feature scales and fusion methods are shown in Table 3.

Shown in the table, *A*_*tr*_ is the training accuracy and *A*_*te*_ is the test accuracy.

It can be seen from Table 5 that the highest test accuracy under the two scales is relatively close, the channel weights under the two scales are suitable for fusion by the linear addition method; the channel weights under the three scales are suitable for selecting the splicing fusion method. Splicing the selection of the splicing coordinate axis in the fusion will affect the accuracy by about 1%. At this time, splicing with the second coordinate axis (cat1) can achieve higher accuracy. In addition, applying the sigmoid function to the output channel weight of the splicing fusion will significantly degrade the classification performance, because the sigmoid function greatly limits the range of values for the channel weights learned by the convolutional layers.

### 4.4. Experiments Based on ResNet34

#### 4.4.1. Comparison and Analysis of msSE-ResNet34 and Other Networks

**Figure 6:**
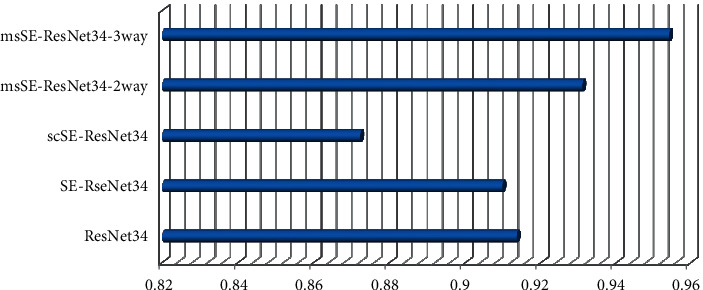
Comparison of AUC between msSE-ResNet34 and other networks.

The experimental results of each network on the test set in the experiment are shown in Table 5, and the ROC curve is shown in Figure 6. As shown in Table 6, all the networks in the experiment are different from the test set magnification of pathological images the multiscale channel recalibration model can make the relationship between channels more accurately captured. Experiments on pathological images of different magnifications prove that msSE-esNet34 can be effectively applied to pathological images of different magnifications classification tasks. In Section 4.4.2, comparison and analysis of msSEResNet34 with different number of feature scales and fusion methods the experimental results of msSE-ResNet34 with different number of feature scales and fusion methods are shown in Table 7.

From Table 7, it can be observed that the highest test accuracy of msSE-ResNet under two different scales is only 0.81% different, with two classification results.

As can be seen from Table 5, with the deepening of the number of ResNet layers, the test accuracy of most networks in the experiment has been greatly improved. SEResNet34 achieved a test accuracy of 87.36%, which is higher than 86.47% of Res-Net34. Applied to the semantic segmentation task of brain MRI images, the experimental results show that the spatial recalibration model does not perform well on the breast cancer pathological image classification task. The test accuracy of msSE-ResNet34-3way rises to the highest 88.87%, 2 the test accuracy of the network at 1 scale is improved to 88.06%.

Table 6 shows the classification results of all networks related to magnification. It can be seen that the accuracy and accuracy of msSE-ResNet34-3way have been greatly improved at all magnifications, especially at 40 times and can reach a maximum of 90.1% at 400 times. The classification accuracy of msSE-ResNet34-2way has been steadily improved at various magnifications. In the experiments under all magnifications, msSEResNet34 is superior to other comparison networks in both precision and accuracy.

The aforementioned experiments demonstrate that msSEResNet34 can best utilize the rich feature details in the deeper network and that the multiscale channel refinement model can enable a more accurate correlation between channels with the intensifying of the network layers. Experiments on pathological images with multiple magnifications demonstrate that msSE-ResNet34 can be effectively applied to the classification task of pathological images with different magnifications.

#### 4.4.2. Comparison and Analysis of msSEResNet34

msSEResNet34 with the different number of feature scales and fusion methods the experimental results of msSE-ResNet34 with different number of feature scales and fusion methods are shown in Table 7.

It can be seen from Table 8 that the highest test accuracy of msSE-ResNet under two different scales is only 0.81% different, and the performance of additive fusion under two scales is better than that under three scales.

**Figure 7:**
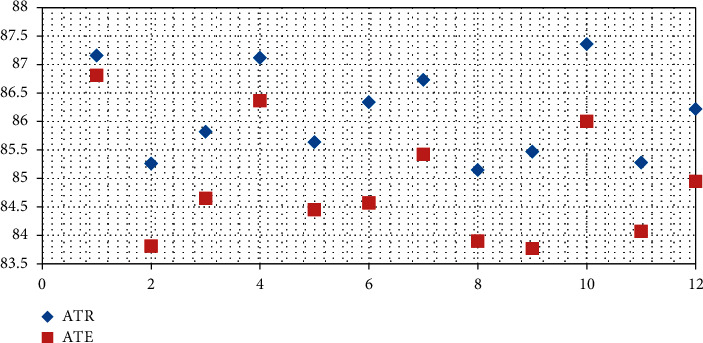
Comparison of classification results of each fusion method under different number of feature scales.

The results obtained in Figure 7, when the network using maximum fusion and splicing fusion at 3 scales is better than 2 scales in classification accuracy. Similar to the conclusion obtained in the experiment based on ResNet18, when there are two scales of features, the addition and fusion of the performance of the method is better than other nonlinear fusion methods, and the splicing fusion method or the maximum fusion method should be preferentially selected under the three scales. The results differ by only about 0.5%, and high classification accuracy can be achieved.

## 5. Conclusion

The classification job of breast cancer pathology pictures is investigated in this research, and a multiscale channel recalibration model called msSE is proposed, as well as a convolutional neural network called msSE-Res-Net using Res-Net as the network architecture. The fusion of feature weights learned at different scales may significantly increase the dependability of the feature channel weight learning process. Multiscale features can enrich the features in the network information and improve feature usage. The BreaKHis dataset experiments reveal that msSE-ResNet with multiscale channel recalibration outperforms SE-ResNet with a single feature scale, as well as the network framework ResNet and the model with spatial and channel recalibration. scSE-ResNet results. The experimental findings on breast cancer pathology pictures at various magnifications demonstrate that the developed msSE-ResNet can be utilized for different magnifications since both the training set and the test set of the network include breast cancer pathology images at different magnifications. Breast pathological pictures with multiples have strong resilience and may be used to classify breast cancer pathological images more effectively. Furthermore, further study is needed into the selection of the compression ratio in the channel recalibration model, as well as the link between multiscale channel recalibration and classification accuracy for convolutional layers at various places in the convolutional neural network.

Various computer vision and machine learning algorithms have been employed for assessing pathological pictures at a microscopic precision as a result of the development of digital imaging methods over the past ten years. These methods could assist in automating some of the problematic workflow-related duties in the diagnostic system. For application in clinical settings, a reliable and effective image processing method is required. Regrettably, conventional methods fall short of expectations. As a result, we are still a long way from using automated breast cancer screening based on histological pictures in clinical settings. These methods, despite their great success in medical imaging, require a lot of label data, which is still lacking in this field of applications for a variety of reasons. Most importantly, annotating a dataset is quite expensive and needs a great deal of knowledge. Future research can focus on issues such as cell overlapping and uneven color distribution in pathological pictures of breast cancer created using various staining techniques.

## Figures and Tables

**Table 1:** Distribution of pictures under different magnifications and categories.

Gain	Number of tumor images		
	Benign	Malignant	Total
40 Times	750	1644	2394
100 Times	773	1725	2498
200 Times	748	1668	2416
400 Times	706	1479	2184

**Table 2:** Comparison of msSE-ResNet18 and other networks' categorization outcomes.

Model	*A*_*cc*_/%	AUC
ResNet18	84.53	0.8878
SE-ResNet18	83.56	0.8791
scSE-ResNet18	83.90	0.8677
msSE-ResNet18-2way	86.81	0.9266
msSE-ResNet18-3way	86.00	0.9107

**Table 3:** Comparison of magnification-related categorization results for all networks.

Model	Number
ResNet18	1
SE-ResNet18	2
scSE-ResNet18	3
msSE-ResNet18-2way	4
msSE-ResNet18-3way	5

**Table 4:** Comparison of magnification-related classification results for all networks.

Model	40 Times			100 Times			200 Times			400 Times		
	*A*_*cc*_	*P*_*r*_	*R*	*A*_*cc*_	*P*_*r*_	*R*	*A*_*cc*_	*P*_*r*_	*R*	*A*_*cc*_	*P*_*r*_	*R*
1	0.822	0.845	0.907	0.836	0.836	0.921	0.864	0.868	0.947	0.875	0.864	0.967
2	0.826	0.820	0.956	0.862	0.861	0.953	0.867	0.862	0.962	0.879	0.865	0.973
3	0.805	0.808	0.941	0.836	0.845	0.935	0.870	0.866	0.962	0.824	0.837	0.918
4	0.862	0.890	0.912	0.862	0.884	0.921	0.880	0.887	0.947	0.889	0.889	0.957
5	0.829	0.856	0.902	0.868	0.878	0.940	0.874	0.905	0.913	0.882	0.884	0.951

**Table 5:** Comparison of categorization results of fusion methods under different number of feature scales.

Number of scales	Fusion method	*A*_*tr*_%	*A*_*te*_%
2	Add	85.42	85.07
2	Max	83.55	82.13
2	Cat1(sign)	84.10	82.96
2	Cat1	85.38	84.64
2	Cat2(sign)	83.93	82.76
2	Cat2	84.61	82.88
3	Add	85.00	83.71
3	Max	83.45	82.22
3	Cat1(sign)	83.76	82.09
3	Cat1	85.61	84.28
3	Cat2(sign)	83.57	82.39
3	Cat2	84.50	83.25

**Table 6:** Comparison of categorization results between msSE-ResNet34 and other networks.

Model	*A*_*cc*_%	AUC
ResNet34	86.47	0.9135
SE-RseNet34	87.36	0.9097
scSE-ResNet34	83.96	0.8722
msSE-ResNet34-2way	88.06	0.9308
msSE-ResNet34-3way	88.87	0.9541

**Table 7:** Comparison of magnification-related classification results for all networks.

Model	40 Times			100 Times			200 Times			400 Times		
	*A*_*cc*_	*P*_*r*_	*R*	*A*_*cc*_	*P*_*r*_	*R*	*A*_*cc*_	*P*_*r*_	*R*	*A*_*cc*_	*P*_*r*_	*R*
1	0.822	0.845	0.907	0.836	0.836	0.921	0.864	0.868	0.947	0.875	0.864	0.967
2	0.826	0.820	0.956	0.862	0.861	0.953	0.867	0.862	0.962	0.879	0.865	0.973
3	0.805	0.808	0.941	0.836	0.845	0.935	0.870	0.866	0.962	0.824	0.837	0.918
4	0.862	0.890	0.912	0.862	0.884	0.921	0.880	0.887	0.947	0.889	0.889	0.957
5	0.829	0.856	0.902	0.868	0.878	0.940	0.874	0.905	0.913	0.882	0.884	0.951

**Table 8:** Comparison of classification results of each fusion method under different number of feature scales.

Number of scales	Fusion method	*A*_*tr*_%	*A*_*te*_%
2	Add	86.28	86.30
2	Max	85.65	85.97
2	Cat1(sign)	84.88	85.01
2	Cat1	86.86	86.28
2	Cat2(sign)	85.29	85.26
2	Cat2	87.40	85.90
3	Add	85.89	85.43
3	Max	86.44	86.87
3	Cat1(sign)	85.89	85.77
3	Cat1	86.59	86.36
3	Cat2(sign)	85.69	86.54
3	Cat2	87.29	87.09

## Data Availability

The data can be obtained from the corresponding author upon request.
